# Radiotherapy in addition to systemic therapy reduces the early mortality of angioimmunoblastic T-cell lymphoma

**DOI:** 10.1007/s00277-026-06796-6

**Published:** 2026-01-23

**Authors:** Yaobin Lin, Qiong Lin, Qizhen Xu, Shenghong Shi, Daxin Huang, Gaoda Ju, Shan Liu, Jianyuan Song, Qingliang Lin, Jianwu Chen

**Affiliations:** 1 Department of Radiation Oncology, Fujian Key Laboratory of Intelligent Imaging and Precision Radiotherapy for Tumors (Fujian Medical University), Clinical Research Center for Radiology and Radiotherapy of Fujian Province (Digestive, Hematological and Breast Malignancies), Fujian Medical University Union Hospital, Fuzhou, Fujian China; 2 Fujian Children’s Hospital, College of Clinical Medicine for Obstetrics & Gynecology and Pediatrics, Fujian Medical University, Fuzhou, Fujian China

**Keywords:** AITL, Early mortality, Radiotherapy, SEER

## Abstract

Angioimmunoblastic T-cell lymphoma (AITL) is a rare and aggressive peripheral T-cell lymphoma that is prone to early progression and recurrence and has a poor overall prognosis. Notably, early mortality (EM) and risk factors for AITL are currently unclear. We performed a retrospective analysis of AITL data from 2000 to 2021 in the Surveillance, Epidemiology, and End Results databases. Early death was defined as death within two years from the date of diagnosis. Histograms and pie charts were used to present the distribution of overall early mortality (O-EM) and lymphoma-specific early mortality (LS-EM). Cox regression model was used to screen the risk factors. Cumulative event rate curves were used to analyze the effect of treatment on EM. In total, 2,413 patients diagnosed with AITL were included in this study. Among the deceased patients, the O-EM was 46.6%, with an LS-EM of 39.7%. EM increased significantly with age, was higher among white person than among other racial groups, and was higher among males than among females. Significant independent risk factors for both O-EM and LS-EM included sex, age, SEER historic stage, radiation therapy, and chemotherapy. The combination of chemotherapy and radiotherapy can decrease O-EM and LS-EM rates in males, aged 40–69 years, and patients with localized and regional SEER historic stages. AITL demonstrates elevated EM. However, the integration of radiotherapy with chemotherapy can significantly reduce the EM among male patients aged 40–69 years with SEER historical stage as localized and regional.

**Supplementary information:**

The online version contains supplementary material available at 10.1007/s00277-026-06796-6.

## Introduction

Angioimmunoblastic T-cell lymphoma (AITL) is a subtype of peripheral T-cell lymphoma (PTCL) that constitutes approximately 2% of all non-Hodgkin lymphomas and 15–20% of PTCLs [1]. Patients with AITL exhibit a high mutation frequency in epigenetic regulatory genes (such as TET2, DNMT3A, and IDH2) [2, 3]. These mutations lead to aberrant DNA methylation, driving the remodeling of the tumor microenvironment (TME), which manifests as vascular hyperplasia, expansion of EBV + B cells, and immune dysregulation [4, 5]. The median age at AITL onset was approximately 65 years, and the condition is characterized by an aggressive disease course that predominantly manifests as systemic lymphadenopathy, itchy rashes, and autoimmune phenomena [6]. The prognosis for AITL remains poor, with 5-year overall survival (OS) and progression-free survival rates of 44% and 32%, respectively [7]. Early death has emerged as a significant contributing factor to the unfavorable prognosis of AITL. In the era of precision therapy, early mortality (EM) and causes of AITL have not been well described.

EM is defined as the death of a patient within a specified period following disease diagnosis and represents a significant challenge in the management of hematological malignancies. For newly diagnosed patients with multiple myeloma, the early mortality rate within the first six months ranges from 10% to 25% [8]. In acute myeloid leukemia, the EM rate is 7.1% within one-month post-diagnosis and can reach up to 11% within two months [9, 10]. For diffuse large B-cell lymphoma, the mortality rate within three months of diagnosis was 36.6% [11, 12]. Currently, the definition of EM in lymphoma has been found to vary considerably across different studies, with timeframes ranging from one month to two years, which increases the difficulty of studying early mortality in hematological diseases. Due to the characteristically short median survival of AITL, typically ranging from 15 to 36 months, and combined with our epidemiological research results, we define EM in AITL as death occurring within 24 months of initial diagnosis. This 2-year threshold effectively captures the majority of premature mortality events driven by aggressive disease progression or treatment-related complications, based on longitudinal cohort analyses [13].

Given the low incidence of AITL, there is currently a lack of large-scale clinical studies addressing EM in patients with AITL. Consequently, knowledge of the EM and its associated factors in patients with AITL remains limited. This study aimed to investigate the EM associated with AITL by integrating clinical characteristics and sociodemographic factors from the Surveillance, Epidemiology, and End Results (SEER) multi-center database. This comprehensive analysis may contribute to the identification of the causes of early death in AITL patients and provide a scientific basis for more proactive treatment strategies.

## Materials and methods

### Patients and ethics

We used the SEER database and statistical software (SEER * Stat 8.3.9) to identify and collect data on patients diagnosed with AITL between 2000 and 2021 according to the International Classification of Cancer Diseases. The study inclusion criteria were as follows: ICD-O-3 Hist/behavior = AITL (ICD-O-3–9840) (*N* = 2,685). We excluded patients with a clinical diagnosis or unknown diagnosis (*N* = 12), lack of follow-up data or follow-up duration is less than one month (*N* = 258), or < 18 years (*N* = 2). Supplementary Fig. 1 illustrates the methodology used in this study. Due to the anonymized nature of the SEER data, no ethical approval was required.

Data were extracted for the following variables: years of diagnosis, sex, age, race, primary site, laterality, B symptoms, marital status, SEER historic stage, Ann Arbor stage, surgery, radiation, chemotherapy, second malignant neoplasm (SMN), and household income. In SEER historic stage, “localized” corresponds to “Ann-Arbor stage I”, “Regional” corresponds to “Ann-Arbor stage II”, and “Distant” corresponds to “Ann-Arbor stage III-IV” (Supplementary Table 1).

### Statistical analysis

The primary endpoints of this study were overall early mortality (O-EM), which refers to death within two years of diagnosis, and lymphoma-specific early mortality (LS-EM), which denotes death specifically attributable to lymphoma within two years of diagnosis. The EM distribution was visualized using histograms and pie charts generated using SPSS version 25. Using the SEER database, we identified the age-standardized incidence of AITL based on the 2000 US standard population criteria. Weighted least squares was used to calculate incidences, and cumulative event rate curves were performed and compared by Kaplan–Meier analysis. Using univariate Cox regression analysis, factors (*P* > 0.05) were selected for inclusion in the multivariate analysis.

## Results

### 1. Overall mortality

**Fig. 1:**
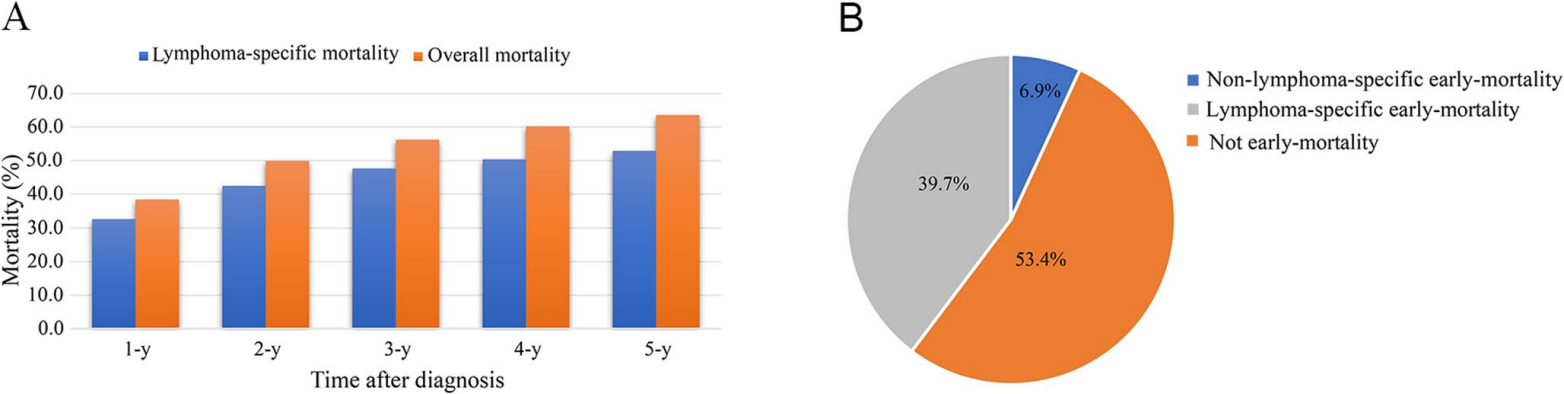
The distribution of causes of death in AITL patients. **A** Comparison of overall and lymphoma-specific mortality in patients with AITL. **B** Distribution of the incidence of lymphoma-specific early mortality, non-lymphoma-specific early mortality, and later mortality among the deceased patients. Abbreviations: AITL: angioimmunoblastic T-cell lymphoma

**Fig. 2:**
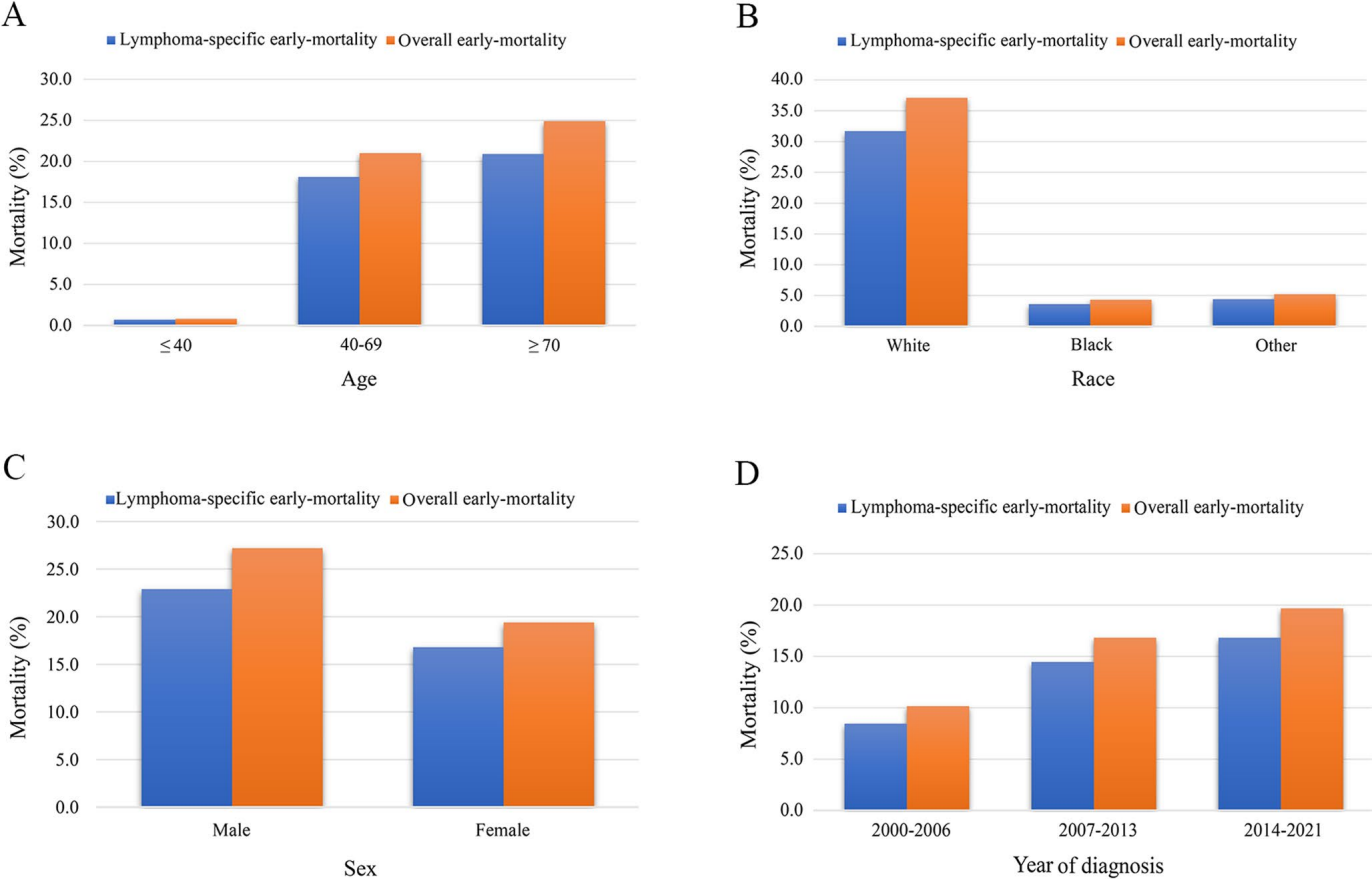
Rates of overall early mortality and lymphoma-specific early mortality in patients with AITL by **A** age, **B** race, **C** sex, and **D** year of diagnosis. Abbreviations: AITL: angioimmunoblastic T-cell lymphoma

In total, 2,413 patients diagnosed with AITL were included in this study. Statistical analysis of overall death revealed that the mortality rates at one, two, three, four, and five years were 38.5%, 50.1%, 56.3%, 60.3%, and 63.8%, respectively (Fig. 1A). The lymphoma-specific mortality at one, two, three, four, and five years were 36.8%, 47.2%, 52.4%, 55.4%, and 58.0%, respectively. The most significant increase in mortality was observed at the 2-year mark. Subsequently, Fig. 1B showed that among the deceased patients, the O-EM rate was 46.6%, of which 39.7% was attributable to LS-EM and 6.9% to non-LS-EM. Furthermore, early death rates exhibited a significant increase with advancing age, were higher among whites compared to other racial groups, and were more prevalent in males than in females (Figs. 2A–C). The year of diagnosis is positively correlated with EM rates (Fig. 2D).

### 2. Clinical features of early death AITL patients

In this study, 1,667 patients died, of whom 1,126 (67.5%) died early and 541 (32.5%) died late (Table 1). There were statistically significant differences between the early death and late death groups in terms of year of diagnosis, sex, age, B symptoms, SEER historical stage, surgery, radiation, chemotherapy, and SMN (all *P* < 0.05). Compared to patients with AITL who died in the late stage, those who died early were more likely to be male, ≥ 70 years old, exhibit B symptoms, and merge with SMN. Patients with AITL who experienced EM were significantly less likely to have undergone surgery (34.4% vs. 40.5%, *P* = 0.015), radiation (2.0% vs. 5.7%, *P* < 0.001), and chemotherapy (73.1% vs. 79.9%, *P* = 0.003). The two cohorts exhibited no significant differences in race, primary site, laterality, marital status, and household income (all *P* > 0.05).

**Table 1:** Comparison of early and late death characteristics of AITL patients

Variable	Total *N*, %	Early Death *N*, %	Late Death *N*, %	*P*
Total	1667(100)	1126(100)	541(100)	
Year of diagnosis				< 0.001
2000–2006	428(25.7)	245(21.8)	183(33.8)	
2007–2013	650(39.0)	406(36.1)	244(45.1)	
2014–2021	589(35.3)	475(42.2)	114(21.1)	
Sex				0.029
Male	942(56.5)	657(58.3)	285(52.7)	
Female	725(43.5)	469(41.7)	256(47.3)	
Age (years)				0.001
≤ 40	27(1.6)	19(1.7)	8(1.5)	
40–69	802(48.1)	507(45.0)	295(54.5)	
≥ 70	838(50.3)	600(53.3)	238(44.0)	
Race				0.184
White	1339(80.3)	895(79.5)	444(82.1)	
Black	141(8.5)	105(9.3)	36(6.7)	
Other/Unknown	187(11.2)	126(11.2)	61(11.3)	
Primary Site				0.976
Lymph nodes	1618(97.1)	1093(97.1)	525(97)	
Non-lymph nodes	49(2.9)	33(2.9)	16(3.0)	
Laterality				0.224
Left unilateral	46(2.8)	33(2.9)	13(2.4)	
Right unilateral	52(3.1)	30(2.7)	22(4.1)	
Bilateral	17(1.0)	14(1.2)	3(0.6)	
Unknown	1552(93.1)	1049(93.2)	503(93.0)	
B symptoms				< 0.001
Present	503(30.2)	382(33.9)	121(22.4)	
Absent	350(21.0)	250(22.2)	100(18.5)	
Unknown	814(48.8)	494(43.9)	320(59.1)	
Marital Status				0.285
Married	987(59.2)	654(58.1)	333(61.6)	
Divorced/separated/Widowed	415(24.9)	293(26.0)	122(22.6)	
Unmarried/Single/Unknown	265(15.9)	179(15.9)	86(15.9)	
SEER historic stage				0.011
Localized	87(5.2)	59(5.2)	28(5.2)	
Regional	114(6.8)	66(5.9)	48(8.9)	
Distant	1405(84.3)	968(86.0)	437(80.8)	
Unknown	61(3.7)	33(2.9)	28(5.2)	
Ann Arbor stage				< 0.001
Stage I-II	154(9.2)	88(7.8)	66(12.2)	
Stage III-IV	1062(63.7)	673(59.8)	389(71.9)	
Unknown	451(27.1)	365(32.4)	86(15.9)	
Surgery				0.015
Yes	606(36.4)	387(34.4)	219(40.5)	
No/Unknown	1061(63.4)	739(65.6)	322(59.5)	
Radiation				< 0.001
Yes	54(3.2)	23(2.0)	31(5.7)	
No/Unknown	1613(96.8)	1103(98)	510(94.3)	
Chemotherapy				0.003
Yes	1255(75.3)	823(73.1)	432(79.9)	
No/Unknown	412(24.7)	303(26.9)	109(20.1)	
SMN				< 0.001
Yes	1130(67.8)	806(71.6)	324(59.9)	
No	537(32.2)	320(28.4)	217(40.1)	
Household income				0.351
< $70,000	459(27.5)	318(28.2)	141(26.1)	
≥ $70,000	1208(72.5)	808(71.8)	400(73.9)	

Abbreviations: *AITL*: Angioimmunoblastic T-cell lymphoma; *SMN*: Second malignant neoplasm; *N*: number

### 3. Cox regression analysis

Next, the predictive factors of O-EM and LS-EM were analyzed. Univariate Cox regression analysis showed that sex, age, marital status, SEER historic stage, Ann Arbor stage, radiation, chemotherapy, and household income differed significantly both for O-EM and LS-EM (all *P* < 0.05, Table 2). Multivariate Cox regression analysis showed that sex, age, marital status, SEER stage history, radiation therapy, chemotherapy, and household income were independent prognostic factors for O-EM and LS-EM (all *P* < 0.05, Table 3). Specifically, male sex, age over 40 years old, divorced/separated/widowed status, SEER historic stage is distant, no radiation, no chemotherapy, and household income less than $70,000 were positively associated with poor O-EM and LS-EM.

**Table 2:** Univariate Cox proportional hazard regression models of O-EM and LS-EM

Variable	O-EM Univariable analysis			LS-EM Univariable analysis	
	Hazard ratio (95% CI)	*P* value		Hazard ratio (95% CI)	*P* value
Year of diagnosis		0.984			0.893
2000–2006	Reference	—		Reference	—
2007–2013	1.006(0.858–1.179)	0.943		1.038(0.873–1.234)	0.671
2014–2021	1.014(0.869–1.183)	0.862		1.038(0.878–1.229)	0.661
Sex					
Male	Reference	—		Reference	—
Female	0.829(0.736–0.933)	0.002		0.850(0.748–0.966)	0.013
Age (years)		< 0.001			< 0.001
≤ 40	Reference	—		Reference	—
40–69	1.879(1.188–2.970)	0.007		1.706(1.065–2.734)	0.026
≥ 70	3.482(2.205–5.498)	< 0.001		3.074(1.921–4.920)	< 0.001
Race		0.089			0.276
White	Reference	—		Reference	—
Black	1.253(1.023–1.533)	0.029		1.197(0.958–1.496)	0.114
Other/Unknown	0.997(0.828–1.202)	0.978		0.990(0.808–1.212)	0.920
Primary Site					
Lymph nodes	Reference	—		Reference	—
Non-lymph nodes	1.143(0.809–1.616)	0.449		1.096(0.748–1.607)	0.638
Laterality		0.248			0.121
Left unilateral	Reference	—		Reference	—
Right unilateral	0.645(0.394–1.058)	0.083		0.579(0.330–1.017)	0.057
Bilateral	1.007(0.539–1.881)	0.983		1.056(0.535–2.084)	0.876
Unknown	0.924(0.654–1.307)	0.657		0.967(0.660–1.419)	0.865
B symptoms		0.092			0.061
Present	Reference	—		Reference	—
Absent	0.837(0.714–0.982)	0.029		0.812(0.683–0.966)	0.018
Unknown	0.931(0.814–1.064)	0.291			
Marital Status		< 0.001			0.001
Married	Reference	—		Reference	—
Divorced/Separated/Widowed	1.328(1.157–1.524)	< 0.001		1.297(1.117–1.507)	0.001
Unmarried/Single/Unknown	0.964(0.817–1.138)	0.667		0.925(0.772–1.108)	0.397
SEER historic stage		0.001			< 0.001
Localized	Reference	—		Reference	—
Regional	1.039(0.731–1.476)	0.832		1.067(0.722–1.577)	0.746
Distant	1.464(1.126–1.904)	0.004		1.580(1.177–2.119)	0.002
Unknown	1.081(0.706–1.655)	0.720		1.029(0.633–1.671)	0.908
Ann Arbor stage		0.001			< 0.001
Stage I-II	Reference	—		Reference	—
Stage III-IV	1.522(1.219–1.900.219.900)	< 0.001		1.687(1.315–2.165)	< 0.001
Unknown	1.395(1.105–1.761)	0.005		1.476(1.136–1.918)	0.004
Surgery					
Yes	Reference	—		Reference	—
No/Unknown	1.088(0.962–1.230)	0.180		1.137(0.994–1.300.994.300)	0.062
Radiation					
Yes	Reference	—		Reference	—
No/Unknown	2.341(1.549–3.539)	< 0.001		2.074(1.359–3.165)	0.001
Chemotherapy					
Yes	Reference	—		Reference	—
No/Unknown	1.584(1.388–1.807)	< 0.001		1.468(1.270–1.698)	< 0.001
SMN					
Yes	Reference	—		Reference	—
No	0.894(0.785–1.017)	0.089		0.869(0.754–1.001)	0.051
Household income					
< $70,000	Reference	—		Reference	—
≥ $70,000	0.836(0.734–0.952)	0.007		0.831(0.722–0.956)	0.010

Abbreviations: *CI*: Confidence interval; *SMN*: Second malignant neoplasm; *O-EM*: overall early mortality; *LS-EM*: lymphoma-specific early mortality

**Table 3:** Multivariate Cox proportional hazard regression models of O-EM and LS-EM

Variable	O-EM Multivariate analysis			LS-EM Multivariate analysis	
	Hazard ratio (95% CI)	*P* value		Hazard ratio (95% CI)	*P* value
Sex					
Male	Reference	—		Reference	
Female	0.707(0.623–0.801)	< 0.001		0.737(0.644–0.844)	< 0.001
Age (years)		< 0.001			< 0.001
≤ 40	Reference	—		Reference	—
40–69	1.963(1.237–3.116)	0.004		1.722(1.070–2.771)	0.025
≥ 70	3.482(2.191–5.535)	< 0.001		2.986(1.852–4.815)	< 0.001
Marital Status		0.003			0.018
Married	Reference	—		Reference	—
Divorced/Separated/Widowed	1.290(1.115–1.493)	0.001		1.257(1.073–1.473)	0.005
Unmarried/Single/Unknown	1.119(0.944–1.325)	0.194		1.068(0.887–1.286)	0.486
SEER historic stage		0.009			0.020
Localized	Reference	—		Reference	—
Regional	1.038(0.728–1.478)	0.838		1.067(0.719–1.582)	0.748
Distant	1.512(1.023–2.237)	0.038		1.456(0.950–2.230)	0.084
Unknown	0.893(0.540–1.476)	0.658		0.809(0.461–1.420)	0.461
Ann Arbor stage		0.678			0.597
Stage I-II	Reference	—		Reference	—
Stage III-IV	1.073(0.713–1.614)	0.737		1.247(0.798–1.947)	0.333
Unknown	1.123(0.765–1.651)	0.553		1.243(0.817–1.892)	0.310
Radiation					
Yes	Reference	—		Reference	—
No/Unknown	1.861(1.225–2.829)	0.004		1.645(1.071–2.526)	0.023
Chemotherapy					
Yes	Reference	—		Reference	—
No/Unknown	1.620(1.407–1.864)	< 0.001		1.515(1.298–1.769)	< 0.001
Household income					
< $70,000	Reference	—			
≥ $70,000	0.821(0.721–0.935)	0.003		0.822(0.714–0.946)	0.006

Abbreviations: *CI*: Confidence interval; *O-EM*: overall early mortality; *LS-EM*: lymphoma-specific early mortality

### 4. Comprehensive predictive factors for early mortality

**Fig. 3:**
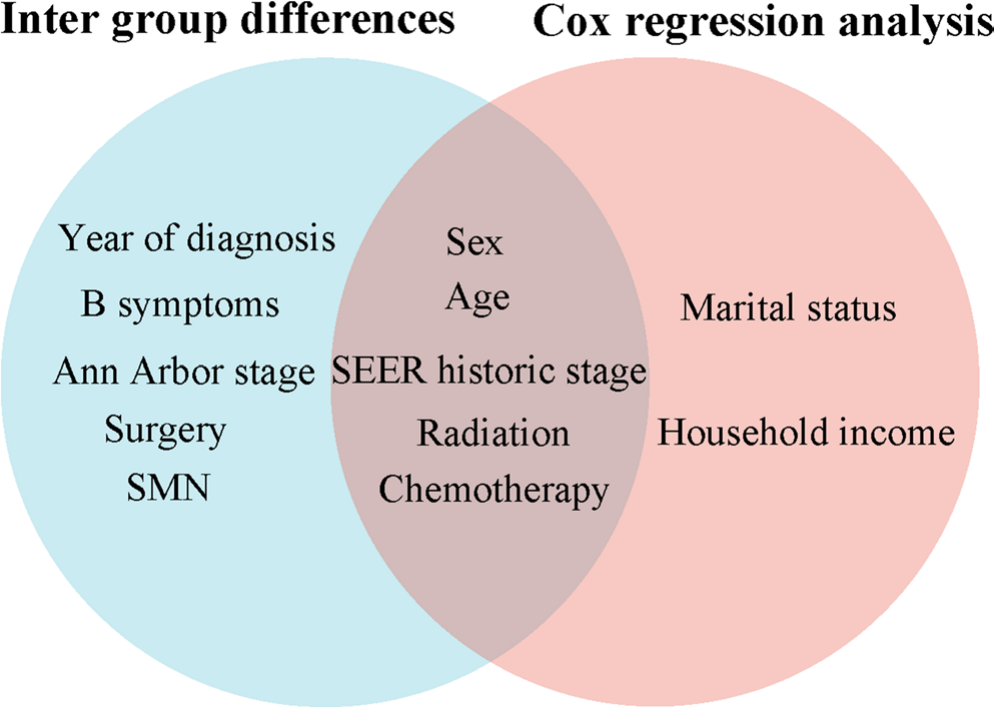
Venn diagram of intergroup distribution difference factors and multivariate Cox analysis independent predictive factors. Abbreviations: SMN: Second malignant neoplasm

Taking the intersection of the intergroup distribution difference factors (Table 1) and independent predictive factors obtained from multivariate Cox analysis (Table 3), the Venn diagram illustrates that sex, age, SEER historic stage, radiation, and chemotherapy were associated with O-EM and LS-EM (Fig. 3).

### 5. The impact of different treatment combinations on early mortality

Evaluation of the survival benefits of chemoradiotherapy (CRT) for selected key predictive indicators.

**Fig. 4:**
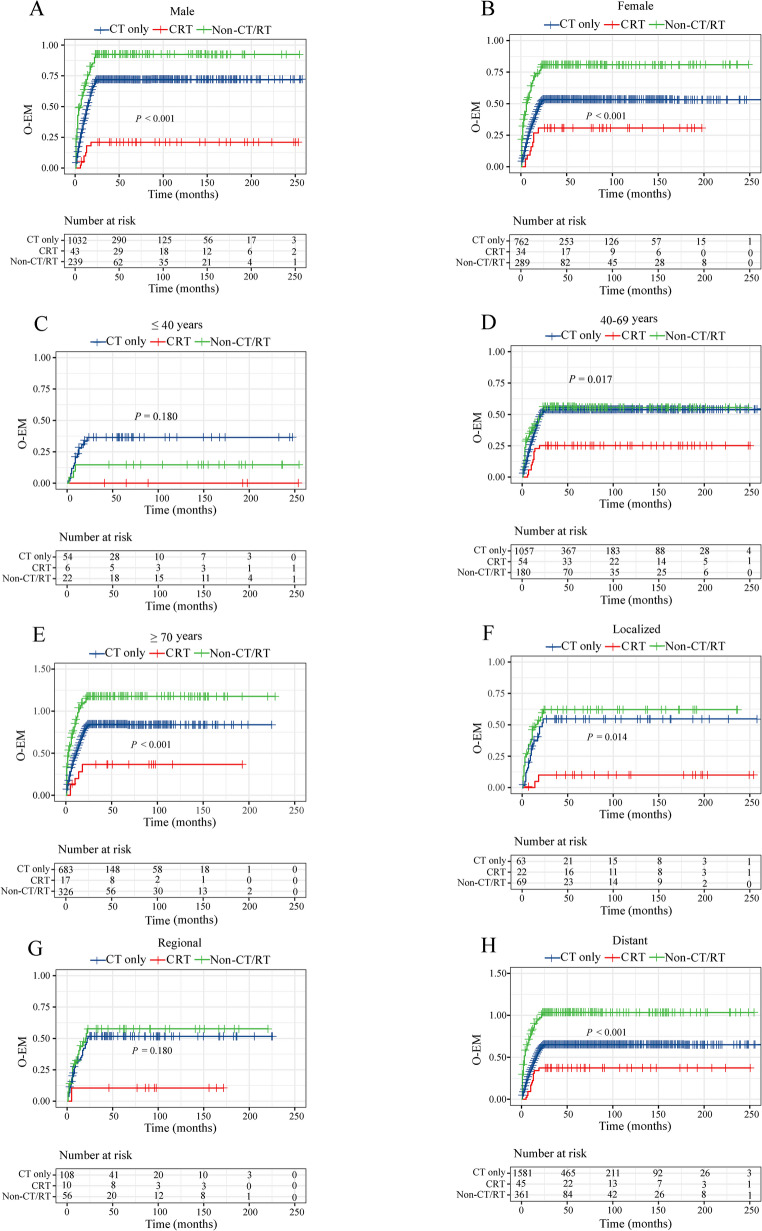
Cumulative event rate curves for the effects of different treatments on overall early mortality: **A** males, **B** females, **C** ≤ 40 years old, **D** 40–69 years old, **E** ≥ 70 years old, **F** localized, **G** regional, and **H** distant. Abbreviations: CT: chemotherapy; RT: radiotherapy; CRT: chemoradiotherapy; Non-CR/RT: no chemotherapy and radiotherapy; O-EM: overall early mortality

**Fig. 5:**
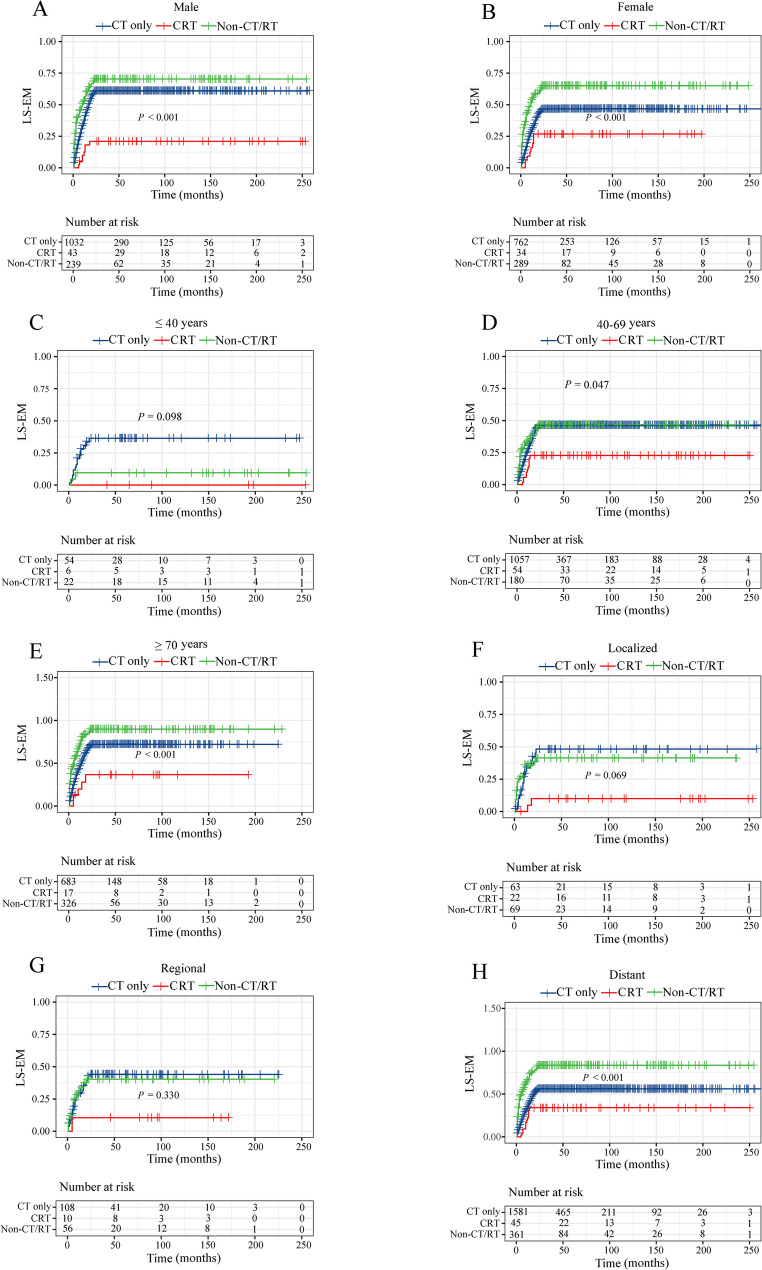
Cumulative event rate curves for the effects of different treatments on lymphoma-specific early mortality: **A** males, **B** females, **C** ≤ 40 years old, **D** 40–69 years old, **E** ≥ 70 years old, **F** localized, **G** regional, and **H** distant. Abbreviations: CT: chemotherapy; RT: radiotherapy; CRT: chemoradiotherapy; Non-CR/RT: no chemotherapy and radiotherapy; LS-EM: lymphoma-specific early mortality

We conducted a comparison of different treatment combinations, as shown in Figs. 4 and 5 and Supplementary Table 2. Compared to chemotherapy, the CRT enhances survival outcomes for O-EM and LS-EM in male, 40–69 years old, and patients with SEER historic stage as localized and regional (all *P* < 0.05). In addition, CRT can also reduce O-EM in patients over 70 years old (*P* = 0.048) and those with distant metastases (*P* = 0.030).

## Discussion

AITL is characterized by high invasiveness and a poor overall prognosis, with early death being a significant risk factor. In this study, we recruited 2,413 patients with primary AITL using multicenter data from the SEER database. Our findings indicate that the O-EM was 46.6%, with an LS-EM of 39.7%. EM increased significantly with age, was higher among white individuals compared to other races, and was higher in males than in females. Screening of the two distribution groups and multivariate Cox analysis identified sex, age, SEER historic stage, radiation, and chemotherapy as independent risk factors for O-EM and LS-EM. Furthermore, the CRT has been found to improve O-EM and LS-EM survival outcomes in certain specific populations, including males, individuals aged 40–69 years, and patients with localized and regional SEER historic stages.

Our study identified that both the overall death and lymphoma-specific death rates of patients with AITL exhibited the most pronounced increases within the first two years. Drawing on previous literature regarding EMs in hematological malignancies [13], we established a cutoff time for EM of two years. Berkman et al. [14]. reported that hematological malignancies had the highest EM (3.1%, 95% CI: 2.9%–3.2%), followed by central nervous system tumors (2.5%, 95% CI: 2.3%–2.8%) and solid tumors (1.0%, 95% CI: 0.9%–1.0%). Despite advancements in pharmacological interventions and ongoing enhancements in diagnostic and therapeutic approaches in recent years, the prognosis of AITL remains unfavorable and is characterized by early disease progression and recurrence [15]. We found that the EM was 50.1% in AITL, which underscores the necessity for heightened attention to EM and its associated risk factors to mitigate the risk of early death.

Prior studies have found that sex, age, and SEER historic stage are independent risk factors for EM in patients with AITL. It is noteworthy that EM was higher in males compared to females, which may be attributed to several factors. (1) There are differences in the immune system between the sexes; females exhibit a higher number of circulating CD4 + T cells than males of the same age [16]. Additionally, the presence of androgens in males promotes the differentiation of CD8 + T cells towards an exhausted state, thereby inhibiting the antitumor immunity of CD8 + T cells [17]. (2) Differences in drug metabolism between males and females have been observed [18], with males exhibiting higher rituximab clearance rates than females, which leads to lower serum concentrations in males [19]. Similarly, males present a higher clearance rate in the pharmacokinetics of obinutuzumab, which affects its efficacy by altering drug concentrations [20]. Age was found to positively associate with EM, which is consistent with previous findings [7] and may be attributed to changes in tumor biology, poor treatment tolerance and compliance, and more complications [21]. An increased risk of EM in distant metastasis patients according to the SEER historical stage was also observed. This overall aligns with the main clinical presentations of AITL: high invasiveness, extensive metastatic potential, poor clinical prognosis, and frequently presenting with distant lymph node metastasis. Approximately 60–80% of patients are diagnosed at an advanced stage (III–IV) [22], which is associated with an increased risk and highlights treatment challenges.

To date, chemotherapy and autologous stem cell transplantation remain the main treatments for AITL; however, our findings revealed that not all patients benefit from chemotherapy. For example, subgroups of patients aged < 70 years and localized/regional SEER historical stage, did not present notable benefits. Interestingly, CRT improved the survival durations of some subgroups, although unfortunately, the efficacy of radiotherapy alone could not be compared (because the number of patients receiving single radiotherapy was too small, which affected the accuracy of the survival analysis). Radiotherapy has been shown to be an effective strategy for treating a variety of lymphomas, especially early-stage disease; however, studies about radiotherapy in AITL are limited. Chen et al. [23]. found that CRT were associated with superior OS compared with chemotherapy alone in patients with PTCL-NOS (5-year OS: 48.3% vs. 27.2%, *P* < 0.05). Meeuwes et al. [24] reported that the 5-year OS of patients with stage I(E) ALCL, AITL and PTCL NOS who received CRT was 72%, while that of patients who only received chemotherapy or radiotherapy was 55% (*P* < 0.01). There is no standardized therapeutic protocol for AITL. According to the NCCN Guidelines (Version 1.2025), the first-line treatment options for AITL include clinical trials, chemotherapy, or chemotherapy combined with radiotherapy. In clinical practice, radiotherapy is less frequently utilized. However, our study suggest that CRT may represent a better treatment strategy for specific patient subgroups, particularly male patients aged ≥ 40 years, potentially conferring a survival advantage by reducing EM.

Radiotherapy in addition to systemic therapy can improve the survival prognosis of AITL, possibly due to the following reasons: (1) Unique pathological features: AITL shows significant vascular proliferation. The use of anti-angiogenic drugs can inhibit the formation of new blood vessels while remodeling the disordered vascular network, increasing oxygen pressure and thereby enhancing the effect of radiotherapy [25, 26]. Furthermore, EBV + B cells are proliferating in the AITL tumor tissues. Nasopharyngeal carcinoma and extranodal NK/T-cell lymphoma (nasal type), which are associated with EBV infection, have been proven to be sensitive to radiotherapy [27, 28]. Radiotherapy can also induce immunogenic death in tumor cells, releasing tumor antigens, promoting the infiltration of CD8 + T cells and dendritic cells, while eliminating Treg cells and M2 macrophages, thereby alleviating the immunosuppressive microenvironment [29, 30]. (2) The synergistic effect of radiotherapy and systemic therapy: Radiotherapy causes DNA double strand breaks through ionizing radiation, while the alkylating agent cyclophosphamide in the CHOP regimen prevents cancer cell replication by disrupting DNA structure [31]. The combination of the two can enhance tumor cell apoptosis through superposition or synergistic effects. AITL is also sensitive to demethylating drugs (azacitidine) and HDAC inhibitors (chidamide) [32]. These drugs may enhance the effect of radiotherapy by reversing the DNA repair mechanism [33, 34]. Furthermore, radiotherapy can induce dysfunction of vascular endothelial cells, detachment from the basement membrane, and induce apoptosis of vascular endothelial cells. During the window of vascular normalization, temporary improvement in tumor vascular permeability promotes the penetration and accumulation of chemotherapy drugs in tumor tissue [35]. (3) Clinical characteristics: Post-chemotherapy residual disease rates in AITL range from 47% to 59% [3, 15, 36]. Radiotherapy can eliminate residual tumor cells through localized high-dose irradiation, thereby reducing recurrence risk. Additionally, AITL patients frequently develop autoimmune manifestations that compromise chemotherapy tolerance. For these patients, radiotherapy offers precise local disease control while minimizing systemic toxicity. The above information indicates that radiotherapy may offer cross-reactivity to chemotherapy and may therefore induce a therapeutic response in patients being refractory to systemic therapy.

Interpreting the findings of this study requires the consideration of several limitations. First, the existence of missing data in the samples inevitably introduces a potential selection bias and accuracy loss. Second, data on tumor recurrence, immunotherapy, targeted therapy, and International Prognostic Index ratings are not record, therefore the above information cannot be included in the study. Third, the database lacked detailed information on treatment modalities, including specific chemotherapy regimens, types and dosages of radiation therapy, and surgical techniques, which impeded further stratification of the dataset based on these variables. Therefore, future prospective, multicenter, large-scale clinical studies are essential to better explore the prognostic factors and treatment recommendations related to AITL.

In conclusion, EM is an important feature of AITL. Variables include sex, age, SEER historic stage, and administration of radiation or chemotherapy were identified as important factors for O-EM and LS-EM. CRT ameliorates adverse outcomes for male patients, aged 40–69 years, and patients with localized and regional SEER historic stages. These findings may assist clinicians in the identification of high-risk individuals and selection of optimal personalized treatment strategies.

## Supplementary information

Below is the link to the electronic supplementary material.

Supplementary Material 1

Supplementary Material 2

Supplementary Material 3

## Data Availability

The datasets used and/or analyzed during the current study are available from the corresponding author on reasonable request.
