# Supplementary material for: Radiotherapy in addition to systemic therapy reduces the early mortality of angioimmunoblastic T-cell lymphoma
Source: Ann Hematol. 2026 Jan 23;105(2):57. doi: 10.1007/s00277-026-06796-6 (PMC12830441; doi:10.1007/s00277-026-06796-6)
Supplement: Supplementary file 1 — Supplementary Material 1 [file 277_2026_6796_MOESM1_ESM.docx]

| **Supplementary Table 1. The distributions of SEER historic stage and Ann-Arbor stage for the included patients.** | | | | | |
| --- | --- | --- | --- | --- | --- |
| **Stage** | Stage I | Stage II | Stage III | Stage IV | Unknown |
| Localized | 114 |  |  |  | 47 |
| Regional |  | 120 |  |  | 55 |
| Distant |  |  | 696 | 632 | 665 |
| Unknown |  |  |  |  | 84 |
