# Supplementary material for: Radiotherapy in addition to systemic therapy reduces the early mortality of angioimmunoblastic T-cell lymphoma
Source: Ann Hematol. 2026 Jan 23;105(2):57. doi: 10.1007/s00277-026-06796-6 (PMC12830441; doi:10.1007/s00277-026-06796-6)
Supplement: Supplementary file 2 — Supplementary Material 2 [file 277_2026_6796_MOESM2_ESM.docx]

| **Supplementary Table 2. The effects of different treatments on early mortality.** | | |
| --- | --- | --- |
| **Variable** | ***P* (**O-EM**)** | ***P* (**LS-EM**)** |
| **Male** |  |  |
| CT only *vs.* CRT | < 0.001 | 0.001 |
| CRT *vs.* Non-CT/RT | < 0.001 | < 0.001 |
| CT *vs.* Non-CT/RT | < 0.001 | 0.003 |
| **Female** |  |  |
| CT only *vs.* CRT | 0.089 | 0.114 |
| CRT *vs.* Non-CT/RT | 0.001 | 0.007 |
| CT *vs.* Non-CT/RT | < 0.001 | < 0.001 |
| **≤ 40 years old** |  |  |
| CT only *vs.* CRT | 0.141 | 0.141 |
| CRT *vs.* Non-CT/RT | 0.354 | 0.455 |
| CT *vs.* Non-CT/RT | 0.155 | 0.069 |
| **40-69 years old** |  |  |
| CT only *vs.* CRT | 0.007 | 0.018 |
| CRT *vs.* Non-CT/RT | 0.005 | 0.016 |
| CT *vs.* Non-CT/RT | 0.331 | 0.411 |
| **≥ 70 years old** |  |  |
| CT only *vs.* CRT | 0.048 | 0.106 |
| CRT *vs.* Non-CT/RT | 0.003 | 0.021 |
| CT *vs.* Non-CT/RT | < 0.001 | < 0.001 |
| **Localized** |  |  |
| CT only *vs.* CRT | 0.010 | 0.019 |
| CRT *vs.* Non-CT/RT | 0.004 | 0.032 |
| CT *vs.* Non-CT/RT | 0.412 | 0.999 |
| **Regional** |  |  |
| CT only *vs.* CRT | 0.045 | 0.034 |
| CRT *vs.* Non-CT/RT | 0.028 | 0.047 |
| CT *vs.* Non-CT/RT | 0.620 | 0.925 |
| **Distant** |  |  |
| CT only *vs.* CRT | 0.030 | 0.066 |
| CRT *vs.* Non-CT/RT | < 0.001 | < 0.001 |
| CT *vs.* Non-CT/RT | < 0.001 | < 0.001 |

| Abbreviations: CT: chemotherapy; RT: radiotherapy; CRT: chemoradiotherapy; Non-CR/RT: no chemotherapy and radiotherapy; O-EM: overall early mortality; LS-EM: lymphoma-specific early mortality. |
| --- |
