# Supplementary material for: Radiotherapy in addition to systemic therapy reduces the early mortality of angioimmunoblastic T-cell lymphoma
Source: Ann Hematol. 2026 Jan 23;105(2):57. doi: 10.1007/s00277-026-06796-6 (PMC12830441; doi:10.1007/s00277-026-06796-6)
Supplement: Supplementary file 3 — Supplementary Material 3 [file 277_2026_6796_MOESM3_ESM.docx]

**
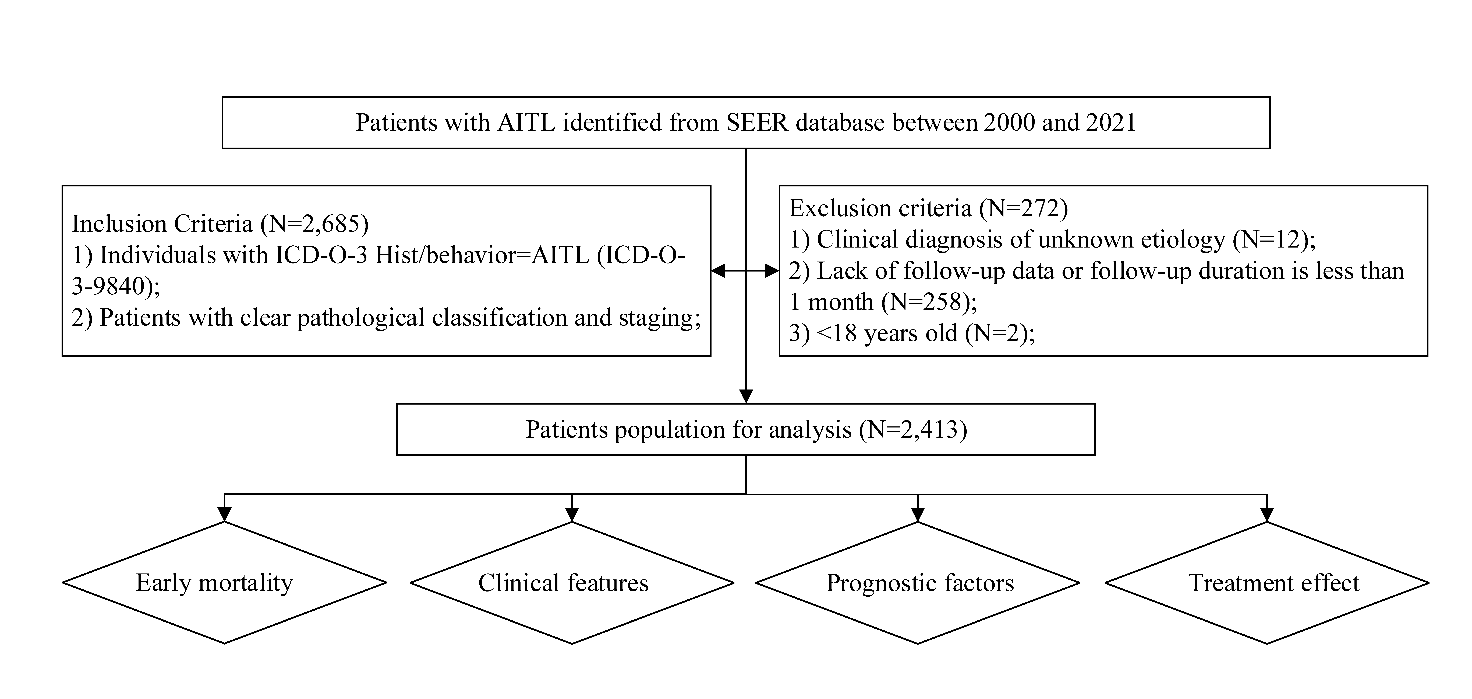
**

**Supplementary Figure 1.** Flow chart of the overall study design.

Abbreviations: AITL: angioimmunoblastic T-cell lymphoma; N: number.
